# Increased attrition of leukocyte telomere length in young adults is associated with poorer cognitive function in midlife

**DOI:** 10.1007/s10654-015-0051-4

**Published:** 2015-06-16

**Authors:** Irit Cohen-Manheim, Glen Michael Doniger, Ronit Sinnreich, Ely Samuel Simon, Ronit Pinchas, Abraham Aviv, Jeremy David Kark

**Affiliations:** Hebrew University-Hadassah Braun School of Public Health and Community Medicine, Ein Kerem, 91120 Jerusalem, Israel; Department of Clinical Science, NeuroTrax Corporation, Bellaire, TX USA; Center for Medical Decision Making, Ono Academic College, Kiryat Ono, Israel; Department of Neurology, Albert Einstein College of Medicine, Bronx, NY USA; Center of Human Development and Aging, Rutgers, New Jersey Medical School, The State University of New Jersey, Newark, NJ USA

**Keywords:** Biomarker, Cognition, Life course epidemiology, Leukocyte telomere length, Telomere attrition, Young adults

## Abstract

Evidence for an association of leukocyte telomere length (LTL) with cognitive function, predominantly in older adults, is inconsistent. No report has examined the association of LTL dynamics (age-specific LTL and its attrition rate) with cognitive function. We aimed to examine the association of LTL dynamics over 13 years in young adulthood with cognitive function in midlife. 497 individuals who had LTL measured at ages 28–32 and 41–46 years were assessed at ages 48–52 for global cognitive function and its five specific component domains with a NeuroTrax computerized test battery. Multivariable regression and logistic models were applied for cognition treated as a continuous and categorical variable, respectively. We found that LTL attrition (adjusted for sex, baseline LTL and potential confounders including socioeconomic variables) was inversely associated with global cognition (standardized β = −.119, *p* = .004) and its component domains: information processing speed (β = −.102, *p* = .024), visual-spatial function (β = −.102, *p* = .017) and memory (β = −.093, *p* = .045), but less so for the attention and executive domains. The multivariable-adjusted odds ratio for low global cognition comparing the upper versus lower thirds of LTL attrition was 2.12 (95 % CI 1.11–4.08, *p* for trend = .023). There was no association of baseline or follow-up LTL with cognition. No effect modification was evident for sex, smoking or inflammatory markers. In conclusion, faster LTL attrition in young adulthood was associated with poorer global and domain-specific cognitive function in midlife, suggesting that more rapid LTL attrition may be predictive of cognitive aging in healthy young adults.

**Electronic supplementary material:**

The online version of this article (doi:10.1007/s10654-015-0051-4) contains supplementary material, which is available to authorized users.

## Introduction

Telomeres, DNA–protein complexes with tandem repeats of 5′-TTAGGG-3′ capping the chromosomal ends, shorten progressively with each somatic cell division, owing to the inability of DNA polymerase to fully replicate the 3′ end of the DNA strand. In cultured somatic cells, telomere length (TL) attrition is enhanced by elevated oxidative stress and, by extrapolation, by inflammation. TL may eventually reach a critical threshold at which a DNA damage signal is generated, resulting in cellular senescence or apoptosis [1]. Consequently, TL has been proposed as a marker of replicative ageing in cultured somatic cells in vitro and presumably in vivo. Growing epidemiological evidence has linked shorter leukocyte TL (LTL) to reduced longevity [2], cardiovascular disease mortality and incidence [3], and atherosclerosis [4]. A shorter LTL has been associated with traits of the metabolic syndrome including hypertension, obesity, insulin resistance, diabetes [1] and low HDL cholesterol [5]. Such aging-, metabolic- and inflammation- related conditions have also been linked to cognitive outcomes [6–10]. Although previous studies have reported an association between shortened LTL and risk of dementia [11], data on LTL and cognitive ageing in non-demented populations is limited and inconsistent [12–18]. Prior studies have typically, but not always [15, 16], focused on older people. We are unaware of reports that have examined the association of LTL attrition at any age with cognitive performance or impairment.

Accordingly, we examined longitudinally the association of LTL dynamics (age-specific LTL and its attrition rate) in young adults with global cognitive function and its specific multi-domain components measured in midlife in a population-based cohort.

## Methods

### Study population

The study sample was based on the Jerusalem Lipid Research Clinic (LRC) Study, a longitudinal, prospective, population-based cohort initiated in 1976–1978 (phase 1). Details regarding sampling and response rates are available [19, 20]. The analysis was undertaken on sample of 497 individuals who completed a cognitive assessment test battery (phase 6, 2009–2011, at ages 48–52) and had LTL measured both in 1989–1991 (phase 4) and 2003–2006 (phase 5). Ethical approval was obtained from the Hadassah Medical Center Helsinki Committee. Signed informed consent was given by all participants.

### Cognitive function

Participants underwent a battery of NeuroTrax computerized cognitive tests (NeuroTrax Corp., Bellaire, TX) selected for the current study to evaluate performance across an array of cognitive domains known to deteriorate during aging (including memory, executive function, visual spatial processing, attention, and information processing speed) in a short administration time of approximately 30 min (0:32 ± 0:04). A global cognitive score reflecting overall cognitive function was computed as the average of the domain scores. Several of these tests are based on common neuropsychological paradigms (including the Benton Visual Retention Test, Brief Visuospatial Memory Test, Tova, Stroop, and subsets of WAIS-III (Wechsler Adult Intelligence Scale, 3rd ed.) and have also been used in clinical settings, as well as in studies of normal aging [21]. Details of the cognitive tests are summarized in Supplementary Appendix 1 and Supplementary Table 1.

Outcome measures that varied with each cognitive domain included accuracy, response time in milliseconds and/or a composite score (the latter given the speed-accuracy tradeoff, computed as accuracy/response time × 100) that has been shown to be valid [22] and reliable [23].

### Leukocyte telomere length (LTL)

Details of the methods of DNA extraction and of LTL measurements using Southern blots have been reported [20, 24]. Briefly, DNA was extracted from the buffy coat of samples stored at −80 °C since 1989–1991 (baseline) and 2003–2006 (follow-up) using Genomic DNA purification kits (Gentra Systems). The integrity of the DNA was verified in all samples. An HinfI/RsaI restriction enzyme combination was used to generate the terminal restriction fragments (TRFs), which were resolved in 5 % agarose gels. The baseline and follow-up TRFs from the same individual were resolved in adjacent lanes of each gel, with duplicate samples run on different gels. The laboratory which received coded samples was blinded as to the identity of the subjects and the order of the baseline and follow-up samples, which varied. The CV for the between-gel duplicates was 2.1 % for the baseline samples and 2.2 % for the follow-up samples [20].

### Covariates

Socio-demographic characteristics consisted of age, sex, origin (father’s country of birth grouped by continent: Europe, Asia, North Africa, and Israel), religiosity (ultra-orthodox, religious, traditional, secular), highest educational attainment (university degree, high school graduate, incomplete high school, elementary), paternal age at birth, sibship size, birth order, early socio-economic position (SEP) (2 measures based on father’s occupation modified from the Israel Central Bureau of Statistics (ICBS), ranked similarly to the British Registrar General’s scale [19], and the Vered Kraus Scale [25]) and adult SEP (2 measures based on the modified ICBS ranking and the MacArthur Scale of Subjective Social Status [26]).

Health behaviors consisted of cigarette smoking (lifetime pack-years), alcohol intake (≤once a week, and a median split of averaged baseline and follow-up number of units per week as 2 dummy variables), and vigorous physical leisure-time activity for at least 20 min causing sweating and shortness of breath (none, either at baseline or follow-up, or at both time-points as 2 dummy variables).

Anthropometric measurements of BMI (in kg/m^2^) and blood pressure were performed at baseline and follow-up. Arterial blood pressure was taken as the mean of 3 seated measures using a standard mercury sphygmomanometer at baseline and an automatic device (Omron M5-I) at follow-up after 5 min of quiet rest. Hypertension was defined as ≥130/85 mm Hg or currently on anti-hypertensive medications.

Plasma glucose, total cholesterol, HDL-C and triglycerides were determined on 12-h fasting samples at both visits by standard enzymatic techniques. LDL-C was computed by Friedewald’s method [27]. Glucose was also measured 2 h after a 75 g glucose challenge. Diabetes was defined by fasting plasma glucose ≥126 mg/dL (7.0 mmol/L) and/or 2 h post-challenge plasma glucose ≥200 mg/dL (11.1 mmol/L) and/or diabetes medication.

Inflammation markers assessed at baseline were plasma concentrations of C-reactive protein (CRP) (by ELISA), fibrinogen (Clauss method), and white blood cell (WBC) count (Beckman Coulter Counter). Serum homocysteine level was determined at baseline (HPLC with fluorometric detection).

Depressive and anxiety symptoms were measured at phase 6 using a translated Hebrew version of the Hospital Anxiety and Depression Scale (HADS) [28] (two 7-item independent subscales).

### Statistical analyses

Cognitive raw outcome measures (i.e., response time, accuracy and composite scores) were z-standardized. Timed measures (response time and response time SD) were multiplied by −1 so that higher values indicate better performance. The z-standardized measures were then averaged to produce five scores, each indexing a different cognitive domain: memory, attention, executive function, visual spatial, and information processing speed. A summary global cognitive score, computed as the average of the 5 domain scores, was treated as the main dependent variable.

Cognitive scores with negatively skewed distributions (global, memory and attention) were Box-Cox [29] power transformed (λ = −.5), i.e., inverse square-root transformed, to achieve an approximately Gaussian distribution, subsequent to reflection (computed by subtracting each value of a negatively skewed score from a constant).

Baseline and follow-up LTL and LTL attrition (the latter computed as the change in telomere bp per year for each individual, accounting for the unequal inter blood draw intervals), were approximately normally distributed (Table 1).

**Table 1:** Characteristics of the study sample

Variables	Total
n	497
Demographic variables	
Age at baseline (year) (range)	30.1 ± .8 (28.1–32.1)^a^
Age at follow-up (year) (range)	43.1 ± .9 (41.2–46.6)
Female (%)	32.8
Origin	
Israel (%)	22.3
Europe (%)	22.7
Asia (%)	29.2
N. Africa (%)	25.8
Education, highest level (%)	
University graduate	32.4
High school graduate	22.1
High school not graduated (9–12 years)	39.4
Elementary school (≤8 years)	6.0
Early SEP (ICBS ranking)^b^	3.8 ± 1.6
Early SEP (Vered Kraus Scale)^c^	44.4 ± 30.2
Adult SEP (ICBS ranking)^b^	2.7 ± 1.2
Adult SEP (MacArthur Scale)^c^	2.0 ± 1.8
Paternal age at birth (year)	33.3 ± 7.2
Birth order	3.0 ± 1.9
Sibship size	4.2 ± 2.8
LTL	
Baseline LTL (bp)	7333 ± 678
Median (IQR)	7295 (6825–7770)
Follow-up LTL (bp)	7001 ± 643
Median (IQR)	6945 (6540–7430)
LTL attrition (bp/year)	25.4 ± 15.3
Median (IQR)	25.2 (16.1–34.6)
Health status variables	
Type II diabetes at baseline (%)	.4
At follow-up (%)	3.0
Hypertension at baseline (%)	4.6
At follow-up (%)	18.5
BMI (kg/m^2^) at baseline	24.7 ± 3.6
At follow-up	27.1 ± 4.4
Depressive symptoms score (HADS 0–21) at endpoint^d^	3.6 ± 2.9
Anxiety symptoms score (HADS 0–21) at endpoint^d^	5.6 ± 3.7
Lifestyle variables	
Leisure-time vigorous activity at baseline (%)^e^	21.6
At follow-up (%)^e^	27.4
Alcohol intake of ≥once/week	
At baseline (%)	39.4
Low intake (units/week)^f^	1.4 ± .5
High intake (units/week)^f^	5.8 ± 2.8
At follow-up (%)	35.4
Low intake (units/week)^f^	1.3 ± .5
High intake (units/week)^f^	6.3 ± 5.8
Pack-years at baseline (whole sample)	4.6 ± 6.5
Among ever smoked	9.4 ± 6.5
Current smokers (%)	38.1
Pack-years at follow-up (whole sample)	8.4 ± 11.8
Among ever smoked	17.3 ± 11.6
Current smokers (%)	31.8
Biochemistry	
Plasma Lipids (mmol/L)	
Total cholesterol at baseline	4.4 ± .8
At follow-up	4.8 ± .8
HDL-cholesterol at baseline	1.0 ± .3
At follow-up	1.1 ± .3
Non-HDL-cholesterol at baseline	3.4 ± .9
At follow-up	3.7 ± .9
LDL-cholesterol^g^ at baseline	2.7 ± .7
At follow-up	3.0 ± .7
Triglycerides at baseline	1.4 ± .9
At follow-up	1.5 ± .9
Fasting plasma glucose (mmol/L) at baseline	5.1 ± .5
At follow-up	5.5 ± .9
Homocysteine (µmol/L) at baseline	12.1 ± 8.5
C-reactive protein (nmol/L) at baseline	21.1 ± 31.2
Fibrinogen (g/L) at baseline	2.3 ± .6
White blood cell count at baseline	6866 ± 1701

Missing data: early SEP (Vered Kraus Scale) (n = 4), adult SEP (ICBS ranking) (n = 5), adult SEP (MacArthur Scale) (n = 11), paternal age at birth (n = 10), birth order (n = 1), depressive symptoms score (n = 4), anxiety symptoms score (n = 4), total cholesterol at follow-up (n = 2), LDL-cholesterol at baseline (n = 7), LDL-cholesterol at follow-up (n = 11), homocysteine (n = 16), c-reactive protein (n = 13), fibrinogen (n = 41), white blood cell count (n = 12)

*LTL* leukocyte telomere length, *bp* base pairs

^a^Mean ± SD (all such values)

^b^A higher value infers a lower SEP. Scale range from 1 to 6

^c^A higher value infers a higher SEP. Vered Kraus scores range from 2.60 to 98.96; MacArthur Scale range from 1 to 10

^d^7-items each scored 0–3. Scale range from 0 to 21. Cronbach’s alphas were adequate at .71 and .785 for the depression and the anxiety subscale, respectively

^e^Defined as exercise for at least 20 min causing heavy breathing and sweating

^f^Low/high intake, according to median split of alcohol intake among consumers of ≥ once/week

^g^Computed by the Friedewald method; not computed for 7 men at baseline and 9 participants (8 men and 1 woman) at follow-up with triglycerides >400 mg/dL (4.52 mmol/L)

Nonparametric Spearman [30] and Pearson correlations between the LTL measures and global function (as well as the five component cognitive domain scores), unadjusted and adjusted for sex, provided similar estimates. Multivariable linear regression models were used to examine the associations between LTL attrition (independent variable) and global function (the dependent variable). These models were repeated separately for each of the 5 cognitive domains to assess to which component(s) the association with global function can be attributed. Regression coefficients are reported as standardized betas (β). Odds ratios (ORs) for the association of LTL attrition with low ranked cognitive function (i.e., scores in the lowest quintile) were computed from logistic models. LTL attrition as a predictor variable was modeled separately in two modes: as a continuous variable and grouped in sex-specific tertiles of change.

All regression models were initially adjusted for sex and baseline LTL. Additional covariates entered in fully-adjusted models included age at cognitive assessment and education identified as important determinants of cognition, plus variables that satisfied the criteria of a univariate association with both LTL attrition and global cognitive score (at *p* < .2), including leisure-time vigorous activity, anxiety, sibship size, birth order and homocysteine level [Box-Cox transformed (λ = −1)]. Not meeting this criterion were origin, religiosity, paternal age at birth, childhood SEP, adult SEP, depression, pack-years of smoking, alcohol intake, BMI, systolic and diastolic blood pressure, insulin resistance (HOMA1-IR), plasma glucose, total cholesterol, HDL-C, non HDL-C, triglycerides, CRP [Box-Cox transformed (λ = 0)], WBC and fibrinogen.

To avoid loss of observations in the multivariable analyses, missing values for birth order (n = 1), homocysteine levels (n = 16) and HADS anxiety score (n = 4 with total score missing and n = 12 with 1 missing item) were replaced by the non-missing mean values.

Effect modification of the LTL-cognitive associations was tested for sex, origin, smoking status at follow-up (current, former, never) and the baseline inflammatory markers (CRP, fibrinogen and WBC) in separate regression models using multiplicative terms. The components of the interaction terms were grand-mean centered to avoid multicollinearity with the main effects.

Statistical analyses were carried out using SPSS v21.0 (IBM Corp., Armonk, NY).

## Results

Characteristics of the study sample are presented in Table 1. Participants were aged 28–32 at baseline and 41–46 at follow-up with a range of 12–16 years of follow-up (13.1 ± .7); 33 % were women, and 55 % were high school or university graduates. Hypertension prevalence increased from 5 to 19 % over the follow-up period, while diabetes prevalence was low at both time points. Mean BMI was high and mean HDL-cholesterol was low (as has been reported [20]), and the prevalence of anxiety was substantial (28 %). Alcohol intake was low as was leisure-time vigorous activity and about one-third of the sample smoked.

LTL characteristics of the study cohort were reported previously [20]. LTL shortening over the 13.1-years mean follow-up period was evident in 481 of the 497 participants. Fifteen individuals (3.0 %) showed elongation, and one showed no change, probably reflecting misclassification due to the combined effects of measurement error of LTL at baseline and follow-up [31]. Mean LTL was 7333 bp at baseline and 7001 bp at follow-up. The average attrition rate was 25.4 ± 15.4 bp/year. Women had longer LTL than men at both baseline and follow-up [by 257 bp (95 % CI 132–382) and 223 bp (95 % CI 104–342), respectively, *p* < .001] as well as a slightly higher LTL attrition rate [by 2.8 bp/year (95 % CI −.1 to 5.7), *p* = .055].

Global cognition, attention, executive and visual spatial mean scores were higher in men than in women (*p* < .03 for all); no sex differences were noted in memory and information processing speed functions (available on request).

Significant inverse Spearman bivariate correlations and sex-adjusted correlations (in light of the sex differences in LTL and cognition) of LTL attrition with cognitive function, but not with baseline and follow-up LTLs, were evident for global cognition, information processing speed, memory and attention scores (the latter with marginal significance) (Table 2). Further adjustment for age at baseline and at follow-up measurement of LTL produced similar estimates. Additional sex-specific analyses showed stronger correlations with LTL attrition in women for the global score (ρ = −.210, *p* = .010) and for information processing speed (ρ = −.174, *p* = .030) and weaker and non-significant correlations in men (available on request), however, the sex interactions were not statistically significant (*p* > .3 for all domains). Consequently, all further analyses were restricted to LTL attrition and were adjusted for sex without including the interaction terms.

**Table 2:** Nonparametric (Spearman) correlations of LTL at ages ~30 years (baseline), ~43 years (follow-up) and average annual LTL attrition with cognitive function in midlife (~50 years)

Model	Global (n = 497)		Attention (n = 497)		Information processing speed (n = 468)		Executive (n = 496)		Visual spatial (n = 495)		Memory (n = 489)	
	ρ	Partial ρ^a^	ρ	Partial ρ^a^	ρ	Partial ρ^a^	ρ	Partial ρ^a^	ρ	Partial ρ^a^	ρ	Partial ρ^a^
1. LTL at baseline	−.021 (.647)	.001 (.977)	−.033 (.462)	−.017 (.705)	−.018 (.704)	−.011 (.808)	−.024 (.590)	.000 (.999)	.022 (.623)	.054 (.231)	−.035 (.441)	−.040 (.377)
2. LTL at follow-up	.017 (.702)	.038 (.399)	−.007 (.874)	.008 (.859)	.024 (.600)	.031 (.509)	−.010 (.830)	.013 (.777)	.049 (.276)	.079 (.080)	−.003 (.941)	−.008 (.867)
3. LTL change/year	−.109 (.015)	−.099 (.028)	−.087 (.054)	−.079 (.078)	−.095 (.040)	−.092 (.047)	−.055 (.224)	−.043 (.338)	−.071 (.115)	−.057 (.206)	−.099 (.029)	−.102 (.025)

*LTL* leukocyte telomere length, *bp* base pairs

*P*-values in parentheses

^a^Adjusted for sex

In regression models adjusted for sex and baseline LTL, poorer performance on global cognitive function and all its five component domains were inversely associated with greater LTL attrition (Table 3, model 1). The associations were strongest for global cognition (standardized β = −.125, *p* = .008), and its components information processing speed (β = −.128; *p* = .009), visual-spatial function (β = −.091; *p* = .051) and memory (β = −.113; *p* = .019).

**Table 3:** Multivariable linear regression of cognitive function in midlife (age ~50 years) on average annual LTL attrition (bp/year) from age ~30 years (baseline) to ~43 years (follow-up)

Models	Global (n = 497)^a^		Attention (n = 497)^a^		Information processing speed (n = 468)		Executive (n = 496)		Visual spatial (n = 495)		Memory (n = 489)^a^	
	Model 1	Model 2	Model 1	Model 2	Model 1	Model 2	Model 1	Model 2	Model 1	Model 2	Model 1	Model 2
	β [*p* value]	β [*p* value]	β [*p* value]	β [*p* value]	β [*p* value]	β [*p* value]	β [*p* value]	β [*p* value]	β [*p* value]	β [*p* value]	β [*p* value]	β [*p* value]
1. LTL attrition (per SD = 15.4 bp/year)	−.125 [.008]	−.119 [.004]	−.061 [.194]	−.064 [.144]	−.128 [.009]	−.102 [.024]	−.032 [.500]	−.038 [.404]	−.091 [.051]	−.102 [.017]	−.113 [.019]	−.093 [.045]
2. LTL attrition (sex-specific tertiles) (2 df)^b^												
1st tertile	0 [.096]^c^ [.037]^d^	0 [.033]^c^ [.012]^d^	0 [.375]^c^ [.189]^d^	0 [.228]^c^ [.107]^d^	0 [.062]^c^ [.033]^d^	0 [.036]^c^ [.031]^d^	0 [.296]^c^ [.471]^d^	0 [.203]^c^ [.375]^d^	0 [.438]^c^ [.209]^d^	0 [.179]^c^ [.064]^d^	0 [.174]^c^ [.070]^d^	0 [.216]^c^ [.097]^d^
2nd tertile	−.030 [.558]	−.032 [.471]	−.014 [.790]	−.016 [.737]	−.014 [.793]	.003 [.944]	.042 [.420]	.042 [.391]	−.022 [.671]	−.038 [.412]	−.070 [.184]	−.068 [.179]
3rd tertile	−.112 [.037]	−.118 [.012]	−.071 [.187]	−.081 [.106]	−.121 [.032]	−.111 [.031]	−.040 [.460]	−.046 [.367]	−.067 [.208]	−.090 [.065]	−.098 [.071]	−.087 [.099]

Model 1: Adjusted for sex and LTL at baseline

Model 2: Fully-adjusted model: age at cognitive assessment, sex, education, leisure-time vigorous activity, sibship size, birth order, plasma homocysteine, anxiety, and LTL at baseline. Reference level of dummy variables: sex, females; educational level, elementary (<9 years); leisure-time vigorous activity, none. Age, anxiety, sibship size, birth order and homocysteine, all introduced as continuous/interval variables

β = standardized regression coefficient

^a^Box-Cox transformed cognitive z-scores (λ = −.5)

^b^Tertile cutoffs of 18 and 31 bp/year in men and 22 and 33 bp/year in women

^c^F test *p* value for the LTL attrition tertiles introduced as dummy variables (2 df)

^d^Test for trend for the LTL attrition tertiles introduced as an ordinal variable (1 df)

In fully adjusted regression models, the associations persisted: an increment of 1 SD of telomere bps shortening per year (15.4 bp/year) was independently associated with poorer scores for global cognition (standardized β = −.119, *p* = .004), and for the information processing speed (β = −.102; *p* = .024), visual-spatial (β = −.102; *p* = .017) and memory domains (β = −.093; *p* = .045) (Table 3, model 2). We repeated the analyses with further adjustment for childhood and adult measures of SEP due to their association with cognitive function; the associations persisted (available on request). To assess possible effects of inflammation, we also included the 3 markers measured at ages 28–32: CRP, fibrinogen and the WBC count; here too the findings persisted (available on request).

Using logistic models to predict low ranked cognition, i.e., the lower fifth of cognitive function, LTL attrition treated as a continuous variable predicted global cognition and the domain of information processing speed. When the extreme tertiles of LTL attrition were compared, the odds ratios for the upper versus the lower thirds were 2.12 (95 % CI 1.11–4.08) and 1.99 (95 % CI 1.03–3.84) for global cognition and information processing speed, respectively (*p* for trend = .023 and .039, respectively) (Table 4, model 2).

**Table 4:** Multivariable logistic regression of low ranked cognitive function in midlife (age ~50 years) on average annual LTL attrition (bp/year) from age ~30 years (baseline) to ~43 years (follow-up)

Models	Global (n = 497)		Attention (n = 497)		Information processing speed (n = 468)		Executive (n = 496)		Visual spatial (n = 495)		Memory (n = 489)	
	Model 1	Model 2	Model 1	Model 2	Model 1	Model 2	Model 1	Model 2	Model 1	Model 2	Model 1	Model 2
	OR (95 % CI) [*p* value]	OR (95 % CI) [*p* value]	OR (95 % CI) [*p* value]	OR (95 % CI) [*p* value]	OR (95 % CI) [*p* value]	OR (95 % CI) [*p* value]	OR (95 % CI) [*p* value]	OR (95 % CI) [*p* value]	OR (95 % CI) [*p* value]	OR (95 % CI) [*p* value]	OR (95 % CI) [*p* value]	OR (95 % CI) [*p* value]
1. LTL attrition (in bp)	1.013 (.998−1.029) [.088]	1.019 (1.001–1.037) [.043]	1.002 (.986–1.017) [.847]	1.004 (.987–1.021) [.660]	1.022 (1.006–1.039) [.007]	1.027 (1.008–1.046) [.005]	1.002 (.987–1.018) [.791]	1.004 (.987–1.021) [.665]	1.007 (.991–1.023) [.385]	1.013 (.995–1.032) [.166]	1.011 (.996–1.027) [.148]	1.010 (.993–1.027) [.255]
2. LTL attrition (sex-specific tertiles) (2 df)^a^												
1st tertile	1 [.187]^b^ [.081]^c^	1 [.073]^b^ [.023]^c^	1 [.705]^b^ [.794]^c^	1 [.650]^b^ [.531]^c^	1 [.191]^b^ [.069]^c^	1 [.118]^b^ [.039]^c^	1 [.563]^b^ [.497]^c^	1 [.519]^b^ [.371]^c^	1 [.877]^b^ [.610]^c^	1 [.434]^b^ [.246]^c^	1 [.195]^b^ [.242]^c^	1 [.138]^b^ [.307]^c^
2nd tertile	1.14 (.65–2.01) [.656]	1.39 (.73–2.66) [.314]	.86 (.49–1.49) [.582]	.92 (.51–1.67) [.791]	1.34 (.74–2.42) [.337]	1.36 (.711–2.61) [.351]	.90 (.52–1.57) [.716]	.96 (.53–1.73) [.880]	1.10 (.63–1.91) [.748]	1.43 (.76–2.71) [.269]	1.68 (.95–2.94) [.072]	1.86 1.01–3.44) [.047]
3rd tertile	1.65 (.94–2.90) [.084]	2.12 (1.11–4.08) [.024]	1.08 (.62–1.88) [.788]	1.21 (.66–2.21) [.533]	1.74 (.96–3.18) [.069]	1.99 (1.03–3.84) [.041]	1.22 (.70–2.12) [.494]	1.31 (.72–2.38) [.374]	1.16 (.65–2.08) [.611]	1.48 (.76–2.87) [.246]	1.44 (.79–2.59) [.232]	1.43 (.75–2.71) [.314]

Model 1: Adjusted for sex and LTL at baseline

Model 2: Fully-adjusted model: age at cognitive assessment, sex, education, leisure-time vigorous activity, sibship size, birth order, plasma homocysteine, anxiety, and LTL at baseline. Reference level of dummy variables: sex, females; educational level, elementary (<9 years); leisure-time vigorous activity, none. Age, anxiety, sibship size, birth order and homocysteine, all introduced as continuous/interval variables

*OR* odds ratio, *CI* confidence interval. Given in square brackets are the *p*-values

^a^Tertile cutoffs of 18 and 31 bp/year in men and 22 and 33 bp/year in women

^b^F test *p* value for the LTL attrition tertiles introduced as dummy variables (2 df)

^c^Trend test *p* value for the LTL attrition tertiles introduced as an ordinal variable (1 df)

Removal of baseline LTL from the multivariable models did not materially affect the association of LTL attrition with global cognition either in the multiple regression model [standardized β = −.114, *p* = .003 vs. β = −.119, *p* = .004 in the fully adjusted model (Model 2 in Table 3)] or in the multivariable logistic model [odds ratio for low global cognition comparing the upper vs. lower thirds of LTL attrition of 2.20 (95 % CI 1.17–4.13) vs. 2.12 (95 % CI 1.11–4.08) in the model including baseline LTL (Model 2 in Table 4) (*p* for trend = .014 vs. 023)].

There was no evidence for effect modification of the LTL-attrition association with cognitive scores or low ranked cognitive function in the multivariable models (*p*-interaction >.3 for all tested) (not shown).

## Discussion

Cognitive function, notably global cognition and domain-specific information processing speed, visual spatial function and memory, among cognitively intact (non-demented) middle-aged adults were associated with the rate of change of LTL (but not with absolute LTL values) over the young adult life course. These associations persisted when adjusted for the effect of age and a rich array of demographic and socioeconomic factors as well as biochemical and somatic measures previously shown to be associated with LTL and/or with cognitive function. We found no reports that assessed the association of LTL attrition with cognitive function.

The results for baseline and follow-up LTL are consistent with previous studies not reporting an association of LTL measured at one point in time with cognitive outcomes [15, 17]. These studies were based on modest sample sizes (between 190 to 351). One study evaluated older people [17] and the other examined individuals aged 44–49 years [15]. However, other studies have reported an association between LTL and cognitive function [12–14, 16]. LTL was associated with cognitive function in a cross-sectional study of 382 healthy women aged 19–78 of whom 192 were aged <50.6 years [16], and in cross-sectional [12] and prospective [13, 14] studies of people ≥65 years of age. Methodological differences between studies, including the age range of the study samples, the timing of telomere measurement relative to the cognitive assessment (simultaneous or prior to outcome), the instrument used to measure cognition and the method of measurement of LTL, may have contributed to the inconsistent findings across studies.

The finding of an LTL attrition-cognition association might be explained through mechanisms involving increased oxidative stress and inflammation that may not only be limited to neurodegenerative diseases [32], but are also found in normal brain aging [32] concomitantly with decline in cognitive function [33, 34]. Oxidative stress accelerates telomere attrition during somatic cell replication, inflammation increases leukocyte turnover rate (therefore may accelerate LTL attrition) [1], and both are associated with cognitive decline [33, 34]. However, we found no attenuation of the LTL-cognition association by adjustment for the inflammatory variables, and no effect modification. Nonetheless, other measures of inflammation not included in this study, including longitudinal measures, might play a role. Another postulated mechanism is that the shortening of LTL and onset of cognitive decline share a common genetic cause (‘‘aging’’ genes) [14].

We acknowledge several limitations of this study. First, a baseline cognition measure of the cohort was not available to us, thus no direct inference about the role of accelerated LTL attrition on cognitive decline can be drawn. Another limitation is the modest sample size (n = 497); nonetheless, the study had sufficient power (≥80 %) to detect correlations between LTL and cognition of a similar effect size (r ≥ .125) to that of a previous report [16]. Lastly, we cannot exclude the possibility of selection bias due to loss to follow-up. However, our study is not affected by the survival bias common to previous reports of elderly people.

The strengths of this study lie in the longitudinal assessment of LTL, the broad array of objective computerized cognitive measures with millisecond precision [35] compared with previous studies, most of which used paper-and-pencil neuropsychological tests, and the wide range of potential confounders evaluated. We stress that the associations withstood adjustment for childhood and adult measures of SEP, both strong predictors of adult cognitive function. The repeated measurements of LTL and the young age at baseline LTL measurement are novel aspects compared with the bulk of previous research on this topic. Furthermore, we note that most previous studies used quantitative PCR, that is associated with substantially larger measurement error than Southern blots [36] employed in our study. Thus, although our sample size is modest, power to detect differences should be correspondingly greater with Southern blots than with quantitative PCR.

In conclusion, this study is the first to address and identify an association of LTL attrition with cognitive function, a finding that requires confirmation. As detection of biomarkers for cognitive decline and future dementia in healthy young individuals is essential, and as LTL attrition is a non-invasive and readily obtainable measure, future longitudinal studies across the lifespan should evaluate whether between-individual variation in LTL attrition is related to changes in cognitive function and subsequent development of cognitive impairment and dementia.

## Electronic supplementary material

Below is the link to the electronic supplementary material.

Supplementary material 1 (DOC 215 kb)
