# Supplementary material for: Increased attrition of leukocyte telomere length in young adults is associated with poorer cognitive function in midlife
Source: Eur J Epidemiol. 2015 Jun 16;31:147–57. doi: 10.1007/s10654-015-0051-4 (PMC4819924; doi:10.1007/s10654-015-0051-4)
Supplement: Supplementary file 1 — Supplementary material 1 (DOC 215 kb) [file 10654_2015_51_MOESM1_ESM.doc]

**Supplementary Appendix 1**

**Description of the NeuroTrax computerized cognitive test battery included in this study**

Following is a brief description of the cognitive tests and the outcome parameters comprising the cognitive domains included in the current study. *(a) The executive function domain* included composite scores from the Go-NoGo response inhibition test, Stroop interference test at phase III, and Catch Game. The Go-NoGo test is a continuous performance test of simple response time (RT) and response inhibition. In the Stroop interference test, the conflicting information provided by the meaning of a word and the color of its letters leads to a decrement in performance relative to the test phases where there is no conflict. The test includes three phases-phase I (no interference: letter color), phase II (no interference: word meaning), and phase III (interference between letter color and word meaning). The Catch Game is a novel motor test requiring hand-eye coordination, scanning and rapid responses. Participants were instructed to 'catch' a rectangular white object falling vertically from the top of the screen by positioning a green rectangular 'paddle' moving horizontally in the path of the falling object. The executive function domain is an average of composite scores from the Go-NoGo response inhibition test and Stroop interference test at phase III, and mean accuracy for the Catch Game; *(b) The memory domain* included immediate and delayed recognition memory tests for the orientation of simple geometric patterns and symbols. Participants were presented with eight black-and-white drawn pictures of simple common objects and were instructed to remember their orientation. Up to four consecutive repetitions tests were administered, followed by a delayed recognition test following one intervening NeuroTrax test. The memory domain is an average of the accuracy on the four immediate repetitions and the delayed phase of the test; (*c) The information processing speed domain* comprised measures of information processing at increasing levels of complexity. The test was comprised of three blocks of information processing load: single digits (block 1), two-digit arithmetic problems (e.g., 5 – 1, block 2), and three-digit arithmetic problems (e.g., 3 + 2 – 1, block 3). For each of these three blocks, stimuli were presented at three different rates (speed levels), incrementally increasing. Participants were instructed to respond by pressing the left mouse button if the digit or result is less than or equal to 4 and the right mouse button if it is greater than 4. The information processing speed domain is a weighted average of the composite scores in block 1 at speed levels 1 (weighted x0.10) and 3 (x0.2) and in block 2 at levels 1 (x0.30) and 2 (x0.40); *(d) The attention domain* was computed as the average of: the mean RT of the Go-NoGo test, the RTSD of the Go-NoGo, the mean RT of the Stroop interference (phase II) and staged information processing speed (block 1, level 2) test, as well as the accuracy score of the latter (block 2, level 3); *(e) The visual spatial processing domain* assesses abstract spatial ability. Participants were presented with familiar daily scenes containing a red pillar (rectangle) and instructed to imagine standing at the location of the red pillar. Four views of the scene were presented and participants were required to indicate which of the four views corresponds to the view of the scene from the location of the pillar. The visual spatial domain is an accuracy score; *(f) The global cognitive* score was computed as the average of the domain scores, thus reflecting overall cognitive function.

| **Supplementary Table 1**  **NeuroTrax tests: Description, cognitive domains and raw scores of tests applied** | | | | | | | | | | | | | |
| --- | --- | --- | --- | --- | --- | --- | --- | --- | --- | --- | --- | --- | --- |
| **NeuroTrax Test** | **Cognitive Domains Tested** | **Outcome Measures** | **Raw Scores** | | | | | | | | | | |
|  |  |  | **Mean** | **SD** | **range** | | | **Median** | **IQR** | | | **10^th^ %ile** | **90^th^ %ile** |
| GO-NOGO  RESPONSE  INHIBITION | Executive Function, Attention |  |  |  |  |  |  |  |  |  |  |  |  |
|  |  | Accuracy | 93.4 | 8.4 | 33.0 | - | 100.0 | 97.0 | 93.0 | - | 97.0 | 87.0 | 100.0 |
|  |  | Average RT | 428.0 | 96.1 | 67.0 | - | 1060.0 | 409.0 | 374.0 | - | 452.0 | 348.8 | 519.6 |
|  |  | RTSD | 108.2 | 69.5 | 41.0 | - | 541.0 | 89.0 | 70.0 | - | 118.0 | 58.0 | 169.3 |
|  |  | Errors of Commission | 1.6 | 1.6 | 0.0 | - | 12.0 | 1.0 | 0.0 | - | 2.0 | 0.0 | 3.0 |
|  |  | Errors of Omission | 0.5 | 1.6 | 0.0 | - | 17.0 | 0.0 | 0.0 | - | 0.0 | 0.0 | 1.0 |
|  |  | RT for Errors of Commission | 336.1 | 144.6 | 7.0 | - | 1156.0 | 302.5 | 268.0 | - | 343.5 | 239.0 | 421.0 |
|  |  |  |  |  |  |  |  |  |  |  |  |  |  |
| NON-VERBAL  MEMORY | Memory |  |  |  |  |  |  |  |  |  |  |  |  |
|  |  | *Immediate Recognition* |  |  |  |  |  |  |  |  |  |  |  |
|  |  | Accuracy, Repetition 1 | 51.8 | 22.8 | 0.0 | - | 100.0 | 50.0 | 38.0 | - | 63.0 | 25.0 | 75.0 |
|  |  | Accuracy, Repetition 2 | 69.1 | 23.9 | 13.0 | - | 100.0 | 75.0 | 50.0 | - | 88.0 | 38.0 | 100.0 |
|  |  | Accuracy, Repetition 3 | 73.7 | 26.6 | 13.0 | - | 100.0 | 75.0 | 50.0 | - | 100.0 | 38.0 | 100.0 |
|  |  | Accuracy, Repetition 4 | 78.4 | 27.9 | 13.0 | - | 100.0 | 88.0 | 63.0 | - | 100.0 | 38.0 | 100.0 |
|  |  | Accuracy, Repetitions 1-4 | 68.4 | 22.6 | 13.0 | - | 100.0 | 75.0 | 54.0 | - | 88.0 | 32.0 | 94.0 |
|  |  | *Delayed Recognition* |  |  |  |  |  |  |  |  |  |  |  |
|  |  | Accuracy | 74.5 | 24.3 | 0.0 | - | 100.0 | 88.0 | 63.0 | - | 94.0 | 38.0 | 100.0 |
|  |  |  |  |  |  |  |  |  |  |  |  |  |  |
| STROOP  INTERFERENCE | Executive Function, Attention |  |  |  |  |  |  |  |  |  |  |  |  |
|  |  | *No Interference: Letter Color [1]* |  |  |  |  |  |  |  |  |  |  |  |
|  |  | Accuracy | 95.9 | 11.2 | 20.0 | - | 100.0 | 100.0 | 100.0 | - | 100.0 | 90.0 | 100.0 |
|  |  | Average RT | 680.1 | 341.5 | 337.0 | - | 4304.0 | 600.0 | 510.0 | - | 732.0 | 435.4 | 968.4 |
|  |  | RTSD | 276.9 | 290.9 | 34.0 | - | 1793.0 | 168.0 | 107.0 | - | 320.0 | 76.0 | 609.2 |
|  |  | *No Interference: Word Meaning [2]* |  |  |  |  |  |  |  |  |  |  |  |
|  |  | Accuracy | 97.7 | 6.1 | 27.0 | - | 100.0 | 100.0 | 100.0 | - | 100.0 | 93.0 | 100.0 |
|  |  | Average RT | 561.5 | 200.4 | 303.0 | - | 2867.0 | 519.0 | 443.0 | - | 627.0 | 391.0 | 761.6 |
|  |  | RTSD | 187.0 | 199.3 | 31.0 | - | 1539.0 | 121.0 | 81.0 | - | 200.0 | 58.0 | 386.8 |
|  |  | *Interference: Letter Color vs. Word Meaning [3]* |  |  |  |  |  |  |  |  |  |  |  |
|  |  | Accuracy | 80.9 | 32.4 | 0.0 | - | 100.0 | 100.0 | 80.0 | - | 100.0 | 7.0 | 100.0 |
|  |  | Average RT | 696.2 | 456.2 | 324.0 | - | 2405.0 | 538.5 | 449.0 | - | 706.0 | 398.4 | 1129.7 |
|  |  | RTSD | 287.9 | 324.5 | 29.0 | - | 1602.0 | 160.0 | 89.0 | - | 335.0 | 61.8 | 670.8 |
|  |  |  |  |  |  |  |  |  |  |  |  |  |  |
| CATCH GAME | Executive Function |  |  |  |  |  |  |  |  |  |  |  |  |
|  |  | Time to Make 1st Move | 572.4 | 135.1 | 359.0 | - | 1498.0 | 546.0 | 486.0 | - | 632.0 | 440.4 | 724.8 |
|  |  | Time to Make 1st Move SD | 156.2 | 66.1 | 59.0 | - | 530.0 | 140.0 | 116.0 | - | 179.0 | 96.0 | 230.4 |
|  |  | Average Direction Changes Per Trial | 0.2 | 0.1 | 0.0 | - | 1.6 | 0.2 | 0.1 | - | 0.3 | 0.1 | 0.4 |
|  |  | Average Error For Missed Catches | 0.3 | 0.2 | 0.0 | - | 1.3 | 0.3 | 0.1 | - | 0.4 | 0.1 | 0.6 |
|  |  | Total Score | 740.0 | 166.3 | 133.0 | - | 1000.0 | 760.0 | 649.0 | - | 840.0 | 513.0 | 920.0 |
|  |  |  |  |  |  |  |  |  |  |  |  |  |  |
| STAGED INFORMATION  PROCESSING SPEED | Attention,  Information Processing Speed |  |  |  |  |  |  |  |  |  |  |  |  |
|  |  | *SINGLE DIGIT* |  |  |  |  |  |  |  |  |  |  |  |
|  |  | *Slow Speed [1.1]* |  |  |  |  |  |  |  |  |  |  |  |
|  |  | Accuracy | 97.4 | 9.7 | 10.0 | - | 100.0 | 100.0 | 100.0 | - | 100.0 | 90.0 | 100.0 |
|  |  | Average RT | 687.7 | 158.4 | 404.0 | - | 1733.0 | 656.0 | 588.5 | - | 749.0 | 526.8 | 861.4 |
|  |  | RTSD | 160.2 | 111.3 | 39.0 | - | 814.0 | 127.0 | 95.0 | - | 174.0 | 75.0 | 297.0 |
|  |  | *Medium Speed [1.2]* |  |  |  |  |  |  |  |  |  |  |  |
|  |  | Accuracy | 96.9 | 7.9 | 30.0 | - | 100.0 | 100.0 | 100.0 | - | 100.0 | 90.0 | 100.0 |
|  |  | Average RT | 603.7 | 112.6 | 381.0 | - | 947.0 | 583.0 | 524.0 | - | 655.5 | 484.6 | 757.8 |
|  |  | RTSD | 139.6 | 89.4 | 26.0 | - | 659.0 | 114.0 | 83.0 | - | 162.0 | 60.0 | 259.2 |
|  |  | *Fast Speed [1.3]* |  |  |  |  |  |  |  |  |  |  |  |
|  |  | Accuracy | 94.4 | 10.0 | 20.0 | - | 100.0 | 100.0 | 90.0 | - | 100.0 | 80.0 | 100.0 |
|  |  | Average RT | 582.3 | 90.0 | 404.0 | - | 932.0 | 566.0 | 522.5 | - | 622.5 | 487.0 | 687.4 |
|  |  | RTSD | 119.4 | 56.6 | 41.0 | - | 344.0 | 107.0 | 83.0 | - | 136.0 | 65.0 | 182.4 |
|  |  | *TWO-DIGIT ARITHMETIC* |  |  |  |  |  |  |  |  |  |  |  |
|  |  | *Slow Speed [2.1]* |  |  |  |  |  |  |  |  |  |  |  |
|  |  | Accuracy | 92.4 | 13.7 | 0.0 | - | 100.0 | 100.0 | 90.0 | - | 100.0 | 80.0 | 100.0 |
|  |  | Average RT | 1232.8 | 272.6 | 690.0 | - | 2488.0 | 1201.0 | 1028.0 | - | 1388.0 | 920.0 | 1622.8 |
|  |  | RTSD | 319.3 | 139.0 | 93.0 | - | 899.0 | 288.0 | 213.0 | - | 406.0 | 165.2 | 527.0 |
|  |  | *Medium Speed [2.2]* |  |  |  |  |  |  |  |  |  |  |  |
|  |  | Accuracy | 93.7 | 10.9 | 50.0 | - | 100.0 | 100.0 | 90.0 | - | 100.0 | 80.0 | 100.0 |
|  |  | Average RT | 974.8 | 195.9 | 544.0 | - | 1656.0 | 953.0 | 835.5 | - | 1083.0 | 737.0 | 1242.0 |
|  |  | RTSD | 230.0 | 89.4 | 63.0 |  | 510.0 | 217.0 | 164.0 |  | 276.5 | 126.0 | 358.0 |
|  |  | *Fast Speed [2.3]* |  |  |  |  |  |  |  |  |  |  |  |
|  |  | Accuracy | 73.5 | 23.7 | 0.0 | **-** | 100.0 | 80.0 | 60.0 | **-** | 90.0 | 40.0 | 100.0 |
|  |  | Average RT | 795.2 | 160.6 | 485.0 | - | 1280.0 | 760.0 | 680.3 | - | 868.0 | 627.5 | 1017.0 |
|  |  | RTSD | 192.5 | 96.0 | 37.0 | - | 680.0 | 168.0 | 130.0 | - | 224.5 | 99.0 | 319.8 |
|  |  | THREE-DIGIT ARITHMETIC |  |  |  |  |  |  |  |  |  |  |  |
|  |  | *Slow Speed [3.1]* |  |  |  |  |  |  |  |  |  |  |  |
|  |  | Accuracy | 84.4 | 16.1 | 20.0 |  | 100.0 | 90.0 | 80.0 | - | 100.0 | 60.0 | 100.0 |
|  |  | Average RT | 1541.8 | 293.3 | 613.0 |  | 2447.0 | 1512.0 | 1335.0 | - | 1706.0 | 1201.6 | 1947.0 |
|  |  | RTSD | 461.7 | 162.2 | 97.0 |  | 1369.0 | 435.0 | 353.5 | - | 534.0 | 287.8 | 652.0 |
|  |  | *Medium Speed [3.2]* |  |  |  |  |  |  |  |  |  |  |  |
|  |  | Accuracy | 70.2 | 23.3 | 10.0 | - | 100.0 | 80.0 | 60.0 | - | 90.0 | 30.0 | 100.0 |
|  |  | Average RT | 1314.3 | 261.3 | 651.0 | - | 2123.0 | 1271.5 | 1129.0 | - | 1460.8 | 1017.7 | 1745.4 |
|  |  | RTSD | 341.4 | 174.5 | 40.0 | - | 1133.0 | 294.5 | 223.8 | - | 387.3 | 175.0 | 629.5 |
|  |  | *Fast Speed [3.3]* |  |  |  |  |  |  |  |  |  |  |  |
|  |  | Accuracy | 49.2 | 22.8 | 10.0 | - | 100.0 | 50.0 | 20.0 | - | 70.0 | 18.5 | 80.0 |
|  |  | Average RT | 931.8 | 244.0 | 196.0 | - | 1332.0 | 882.0 | 740.5 | - | 1225.8 | 651.6 | 1275.1 |
|  |  | RTSD | 310.9 | 218.2 | 16.0 | - | 797.0 | 217.0 | 132.0 | - | 575.5 | 97.0 | 651.5 |
|  |  |  |  |  |  |  |  |  |  |  |  |  |  |
| VISUAL SPATIAL  PROCESSING | Visual Spatial |  |  |  |  |  |  |  |  |  |  |  |  |
|  |  | Accuracy | 58.6 | 19.2 | 13.0 | - | 100.0 | 63.0 | 44.0 | - | 75.0 | 31.0 | 81.0 |
|  |  |  |  |  |  |  |  |  |  |  |  |  |  |
| %ile-Percentile  RT-Response time  SD- Standard deviation | | | | | | | | | | | | | |
